# Expanding the genetic landscape of endometriosis: Integrative -omics analyses implicate key genes and pathways in a multi-ancestry study of over one million women

**DOI:** 10.21203/rs.3.rs-8116602/v1

**Published:** 2025-12-04

**Authors:** Lindsay A Guare, Jagyashila Das, Lannawill Caruth, Ananya Rajagopalan, Alexis T. Akerele, Ben M Brumpton, Tzu-Ting Chen, Leah Kottyan, Yen-Feng Lin, Elisa Moreno, Ashley J Mulford, Marija Simona Dombrovska, Yuan Luo, Vita Rovite, Alan R Sanders, Craig Teerlink, Danielle Candelieri, Noemie Elhadad, Andrew Hill, Gail P. Jarvik, James Jaworski, Julie Lynch, Shinichi Namba, Yukinori Okada, Yue Shi, Yuya Shirai, Jonathan Shortt, Wei-Qi Wei, Chunhua Weng, Yuji Yamamoto, Penn Medicine Biobank, Regeneron Genetics Center, Global Biobank Meta-analysis Initiative, Sinead Chapman, Wei Zhou, Todd Edwards, Suneeta Senapati, Digna R. Velez Edwards, Shefali Setia-Verma

**Affiliations:** 1 Pathology and Laboratory Medicine, University of Pennsylvania, Philadelphia, PA, United States; 2 Genomics and Computational Biology, University of Pennsylvania, Philadelphia, PA, United States; 3 Division of Quantitative Science, Department of Obstetrics and Gynecology, Department of Microbiology, Immunology, and Physiology, Vanderbilt University Medical Center, and Meharry Medical College, Nashville, TN, United States; 4 HUNT Center for Molecular and Clinical Epidemiology, Norwegian University of Science and Technology, Trondheim, Norway; 5 Center for Neuropsychiatric Research, National Health Research Institutes, Maoli County, Taiwan; 6 Cincinnati Children’s Hospital Medical Center, Cincinatti, OH, United States; 7 Endeavor Health, Evanston, IL, United States; 8 Latvian Biomedical Research and Study Centre, Riga, Latvia; 9 Feinberg School of Medicine, Northwestern University, Chicago, IL, United States; 10 VA Informatics and Computing Infrastructure, VA Salt Lake City Healthcare System, Salte Lake City, UT, United States; 11 Biomedical Informatics, Columbia University, New York City, NY, United States; 12 Colorado for Precision Medicine, Aurora, CO, United States; 13 Division of Medical Genetics, University of Washington, Seattle, WA, United States; 14 Division of Epidemiology, Department of Medicine, Vanderbilt University Medical Center, Nashville, Tennessee, United States; 15 Genome Informatics, The University of Tokyo, Tokyo, Japan; 16 Broad Institute, Cambridge, MA, United States; 17 Statistical Genetics, Osaka University, Suita, Osaka, Japan; 18 Biomedical Informatics, Vanderbilt University, Nashville, TN, United States; 19 Division of Epidemiology, Department of Medicine, Vanderbilt University Medical Center, Nashville, TN, United States; 20 Division of Reproductive Endocrinology and Infertility, Department of Obstetrics and Gynecology, Hospital of the University of Pennsylvania, Philadelphia, PA, United States; 21 Division of Quantitative Science, Department of Obstetrics and Gynecology, Department of Microbiology, Immunology, and Physiology, Vanderbilt University Medical Center, Nashville, Tennessee, United States

## Abstract

We report the findings of a genome-wide association study (GWAS) meta-analysis of endometriosis across 14 biobanks worldwide, including 32% non-European patient participants, as part of the Global Biobank Meta-Analysis Initiative (GBMI). Out of 58 total loci (29 previously unreported), the largest meta-analysis accounted for 46 (20 previously unreported). We detected the first genome-wide significant loci (*2p13.3* and *20q13.2*) uniquely driven by the African-ancestry meta-analysis. Our imaging- and surgery-confirmed phenotypes yielded six additional previously unreported loci. Leveraging our large and diverse study population, we observed SNP heritability estimates of 9–13% for all ancestry groups, and 13 loci had at least one variant in the credible set after fine-mapping. Investigating the complex array of endometriosis comorbidities and risk factors revealed 135 genetically correlated phenotypes and 95 with evidence of vertical pleiotropy, including triglycerides and anxiety disorders. We prioritized 35 disease-relevant cellular contexts from the endometrial cell atlas and found 322 examples of differentially expressed genes in cells from donors with endometriosis. Further high-throughput multi-omic analyses implicated a total of 282 genes in endometriosis pathogenesis. Our diverse, comprehensive GWASs, with downstream analyses spanning molecular to phenotypic scales, provide detailed evidence for aspects of endometriosis including the role of immune cell types, Wnt signaling, and cellular proliferation. These interconnected pathways and risk factors underscore the complex, multi-faceted etiology of endometriosis, suggesting multiple targets for precise and effective therapeutic interventions.

## Introduction

Endometriosis, a debilitating condition characterized by the growth of endometrial-like tissues outside of the uterus affects approximately 10% of women of reproductive age worldwide^2^. The precise mechanisms of these lesions are presently unknown. Endometriosis is incurable, leaving patients to manage severe pain^3^ and cope with fertility challenges^4^. Despite its prevalence and impact, the etiology of endometriosis remains poorly understood, hampering efforts to develop effective diagnostic tools and targeted treatments.

Genome-wide association studies (GWASs) have been an important tool for suggesting candidate genes and pathways linked with endometriosis^5–7^. GWASs for endometriosis have uncovered over 40 loci associated with endometriosis risk. However, like most GWASs historically, the previous endometriosis studies have primarily included European ancestry populations and at a smaller scale, East-Asian^8^. While the broad sense heritability of endometriosis has been estimated as 47% via a twin study^9^, previous endometriosis GWASs have only explained about 7% of phenotypic variance with common variants^5,6^ and have fallen short in elucidating the full spectrum of its pathophysiology.

The advent of large-scale biobanks has revolutionized genetic research, offering unique opportunities to conduct well-powered studies across diverse populations^10–13^. As more diverse genomic datasets become available like the Penn Medicine Biobank (PMBB)^14^, the All of Us Research Program (AOU)^15^, and the Million Veterans Program (MVP)^16^, they enhance the discovery of trait loci through increased statistical power and variation enriched in non-European populations^10^. A worldwide consortium of genomic researchers, the Global Biobank Meta-Analysis Initiative (GBMI), was established to facilitate collaboration in GWAS studies on unprecedented scales. In this study, we at the PMBB have collaborated with 12 other biobanks across several countries^15,17–27^ and incorporated publicly-available summary statistics from FinnGen, totaling 14 global datasets. These resources not only enhance statistical power to detect novel associations but also provide rich phenotypic data, enabling more refined analyses. Moreover, the inclusion of diverse ancestries in genetic studies is crucial to aid in fine-mapping and for improving the generalizability of findings and addressing health disparities. This is particularly pertinent for endometriosis, where significant variations in prevalence and clinical presentation have been observed across different ethnic groups^28^.

Beyond GWAS, there are many methods which can be employed *in silico* to investigate other elements of the central dogma, providing further validation of GWAS loci and discovery of additional disease-associated genes. Recent advances in multi-omics technologies have further expanded our ability to translate genetic associations into biological insights. Integration of GWAS results with multi-omic QTL and single-cell datasets using methods such as imputed association studies and Mendelian randomization allows for a more comprehensive understanding of disease mechanisms, potentially identifying novel therapeutic targets and biomarkers. For endometriosis, where the interplay between genetic predisposition and complex tissue-specific processes is central, integrative approaches are especially valuable. Given these considerations, there is a critical need for a large-scale, ancestrally diverse GWAS of endometriosis that incorporates rigorous phenotyping and leverages multi-omics data to provide a more complete picture of the disease’s genetic landscape and underlying biology.

Here, we present the results of a large-scale GWAS meta-analysis that significantly advances our understanding of endometriosis pathophysiology. Our study encompasses a diverse cohort from 14 biobanks worldwide, with over 30% non-European participants, enhancing the generalizability of our findings. We employed a comprehensive phenotyping approach, including not only a wide spectrum of endometriosis presentations but also surgically confirmed and procedure-validated (surgery or imaging) phenotypes. This robust phenotyping strategy enables greater resolution when examining the genetic architecture of endometriosis. Through high-throughput exploration of pleiotropy and integrative multi-omics analyses, we have explored numerous layers of endometriosis pathogenesis. These findings provide unprecedented insights into the risk factors and molecular mechanisms driving this complex disorder, paving the way for improved diagnostics and targeted therapeutic interventions.

## Results

### EHR-Based Phenotyping of Endometriosis in Genetically Diverse Datasets

Of the six case-control definitions (W - Wide, Wex - Wide excluding adenomyosis, PCN.v2 and PCN.v2 - Procedure-Confirmed Narrow [vs (1) all and (2) confirmed controls], and SCN.v1 and SCN.v2 - Surgically-Confirmed Narrow [vs (1) all and (2) confirmed controls]), the largest phenotype, W, comprised 1,063,657 women (31.9% non-EUR), including 50,265 cases. The Wex phenotype, for which several biobanks were omitted, had a total sample size of 615,050 (N_cases_ = 12,262). Sample sizes for the PCN and SCN phenotypes were smaller (Table 1), with summary statistics included from only nine of the 14 biobanks (Supplementary Table 1).

### Multi-Ancestry Wide Phenotype GWAS Meta-Analysis Results

**Figure 1:**
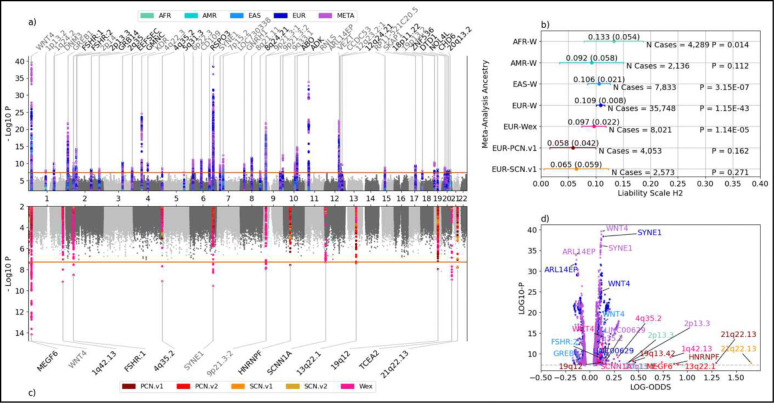
Overview of 11 GWAS meta-analysis results. a) Top left: overlayed Manhattan plots for the five Wide endometriosis phenotype GWASs (multi-ancestry + four single-ancestries). All significant loci are annotated with black text highlighting the previously unreported hits. b) Top right: LD-score regression (using LDSC) heritability estimates for the W GWASs; AMR is excluded because there was not sufficient sample size to use LDSC. c) Bottom left: Overlayed Manhattan plots for the five multi-ancestry GWASs for the other phenotypes. d) Bottom right: volcano plot highlighting variants with an absolute log-odds effect size > 0.1, colored by ancestry group. Phenotype abbreviations: W = wide, Wex = wide excluding adenomyosis, PCN = procedure-confirmed narrow, SCN = surgically-confirmed narrow, .v1 = versus all controls, .v2 = versus confirmed controls. Ancestry abbreviations: AFR = African, AMR = admixed American, EAS = east Asian, EUR = European.

Across the 11 meta-analyses (six multi-ancestry meta-analyses for the different phenotype definitions plus five single-ancestry W meta-analyses), we identified 3,165 unique genome-wide significant variants (P < 5 × 10^−8^), corresponding to 5,049 associations (Supplementary Table 2). These associations were aggregated into 249 linkage disequilibrium (LD) clumps (Supplementary Table 3), which further merged into 104 distinct loci across the 11 meta-analyses (Supplementary Table 4). When we consolidate loci shared between meta-analyses, we arrive at 58 total loci. Study-wide loci shown in Figure 1 are labeled with the most significant corresponding gene from the downstream multi-omic analyses (described below) or the cytoband (Supplementary Table 5). The largest analysis (multi-ancestry W phenotype) resulted in 46 loci associated with endometriosis. Besides those, there were three EUR-specific W loci (*GRB14, ZNF536,* and *CHD6*), totaling 49 W loci annotated in Figure 1a. The two AFR and two of the seven EAS W loci were unique among the single-ancestry GWASs but all were overlapping with signals from the multi-ancestry meta-analyses; meanwhile, the AMR and CSA+SAS W GWASs did not yield any genome-wide significant loci. Of the 49 unique W loci, 26 of the lead SNPs were in LD (R^2^ > 0.1) with the lead SNPs reported in *Rahmioglu et al, 2023*, and an additional three were found within 1 Mb of the previous lead SNPs^6^. The associations between endometriosis and the 20 remaining W loci are reported here for the first time.

Liability scale genome-wide SNP heritability estimates and standard errors (h^2^_lia_) from the W single-ancestry meta-analyses are shown in Figure 1b. The h^2^ estimates and their standard errors were: 0.133 (0.054) for AFR, 0.106 (0.021) for EAS, and 0.108 (0.008) for EUR. Heritability estimates (via the LD-score regression tool, LDSC) for all analyses are provided in Supplementary Table 6. Partitioned LD-score regression (Supplementary Table 7) attributed significant proportions of the phenotypic variance to uterine-specific active enhancers (epigenome map state called “EnhA2”) in EAS (prop_h2_ = 33%, enrichment = 26-fold, P_prop h2_ = 1.2 × 10^−3^) and EUR (prop_h2_ = 20%, enrichment = 15-fold, P_prop h2_ = 6.6 × 10^−4^). Two others explained significant portions of the heritability: ncRNA_gene (non-coding RNA genes) in EAS (prop_h2_ = 21%, enrichment = 1.4-fold, P_prop h2_ = 2.6 × 10^−3^) and TssFlnk (regions flanking transcription start sites) in EUR (prop_h2_ = 7%, enrichment = 1.3-fold, P_prop h2_ = 0.031). Cross-ancestry genetic correlation estimates with Popcorn were under-powered, with none being significantly different from one. The strongest estimate was between EAS and EUR: *ρ*_gi_ = 0.76, P = 0.063 (all genetic impact correlation estimates are available in Supplementary Table 8).

### Precise Phenotype Analyses Replicate Known Loci and Reveal Additional Loci

Across the remaining phenotype meta-analyses, we identified 14 significant loci: eight with Wex, three with PCN.v1, one with both PCN.v1 and SCN.v1, and two with PCN.v2. Three of the eight loci associated with Wex in the multi-ancestry analysis replicate previously reported signals, while the other five are previously unreported: *1q12.13, FSHR:1, 4q35.2, SCNN1A,* and *TCEA2*. All six loci associated with the narrow phenotypes are previously unreported (Figure 1c). The surgically-confirmed phenotype associations displayed the most extreme effect sizes (Figure 1d), including *21q22.13* (rs186666195:T OR_SCN.v1_ = 5.23 and OR_PCN.v1_ = 3.65) and *13q22.1* (rs189908888:T OR_PCN.v2_ = 2.48). The liability scale heritability captured by the EUR Wex GWAS was 0.096 (0.0018). Heritability estimates for the remaining narrow phenotypes were non-significant and are provided in Supplementary Table 6. None of the partitioned heritability estimates for the Wex and narrow phenotypes (EUR-only) reached significance (Supplementary Table 7).

### Fine-Mapping to Detect Causal Variants at GWAS Loci

To characterize shared and population-specific putative causal variants, we conducted statistical fine-mapping (See Methods) on all genome-wide significant regions from multi-population meta-analyses, totaling 63 significant loci in five of the six studies (the SCN.v2 meta-analysis yielded no genome-wide significant results). Among these, 13 loci contained at least one variant with posterior inclusion probability (PIP) greater than 0.5, corresponding to 85 unique causal variants with strong evidence for causality (Supplementary Table 9). The cross-ancestry fine-mapping analyses computed PIPs for each variant across all combinations of ancestry groups included in the overall study. We identify credible sets (CS) for the 13 distinct loci associated with endometriosis as reported in Table 2.

Of the 13 loci with at least one variant in the credible set, nine were from the W multi-population meta-analysis, two were from Wex, and there was one each from PCN.v1 and PCN.v2. Twelve of the credible sets included variants detectable in part due to the incorporation of additional genetic ancestry groups (AMR, AFR, and SAS) previously absent from analyses. In particular, two loci (*FSHR:2* and *ADK.)* derived their credible sets from incorporating four of the five ancestry groups. All PIPs from MESuSiE are available in Supplementary Table 9.

### GWAS Variant-Set Gene Enrichment Analyses

From 11 meta-analyses, we identified 78 associations with 46 unique gene regions that were enriched for significant GWAS variants. Eleven gene regions from MAGMA did not overlap with significant GWAS loci, of which 9 were detected by W analyses. Two of the 9 were significant in both the META and EUR wide phenotype GWASs: *CCHCR1* and *KCTD13*. The other two non-GWAS significant MAGMA hits were for the Wex (*GLB1L3*, P = 3.66 × 10^−7^) and PCN.v1 (*LRRC2*, P = 3.40 × 10^−7^) phenotypes. All significant MAGMA results are reported in Supplementary Table 10.

### Exploring Pleiotropy via Genetic Correlation and Mendelian Randomization

**Figure 2:**
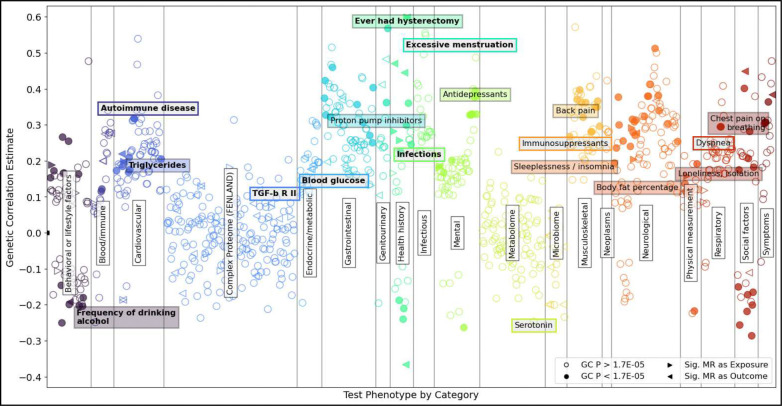
Genetic correlation (GC) and Mendelian randomization (MR) analyses. Each scatter point, colored by category, represents a GC estimate for which the p-value was less than 0.05. Filled shapes indicate a Bonferroni-significant GC value. Triangles indicate significant causal relationships detected by MR. Selected results are highlighted with boxes. A color fill corresponds to a significant GC estimate whereas a colored outline did not reach multiple testing GC significance. Bold text in the annotation boxes indicates a significant MR pair.

Out of 3,065 genetic correlation tests spanning complex trait GWASs from the UK Biobank (UKBB) phenome, the All of Us (AOU) phenome, microbiome, and partial proteome datasets, 890 traits were nominated for bidirectional Mendelian randomization analysis. A total of 135 phenotypes showed significant genetic correlation with endometriosis at a Bonferroni-corrected threshold of P < 1.63 × 10^−5^, including age at first live birth (rg = −0.24), sleeplessness/insomnia (rg = 0.19), headache (rg = 0.30), and antidepressants prescription (rg = 0.39). Evidence for vertical pleiotropy was observed in 95 exposure-outcome pairs, including associations suggestive of endometriosis contributing to risk of hyperlipidemia, prescription of antacids, and reduced levels of Apolipoprotein F and *Bifidobacteriaceae*. Nominated genetic correlation traits and their MR results are shown in Figure 2. Detailed results of the analyses are provided in Supplementary Tables 11 and 12 for genetic correlation and MR, respectively.

### Identifying Disease-Relevant Cell Types and Crucial Genes

**Figure 3:**
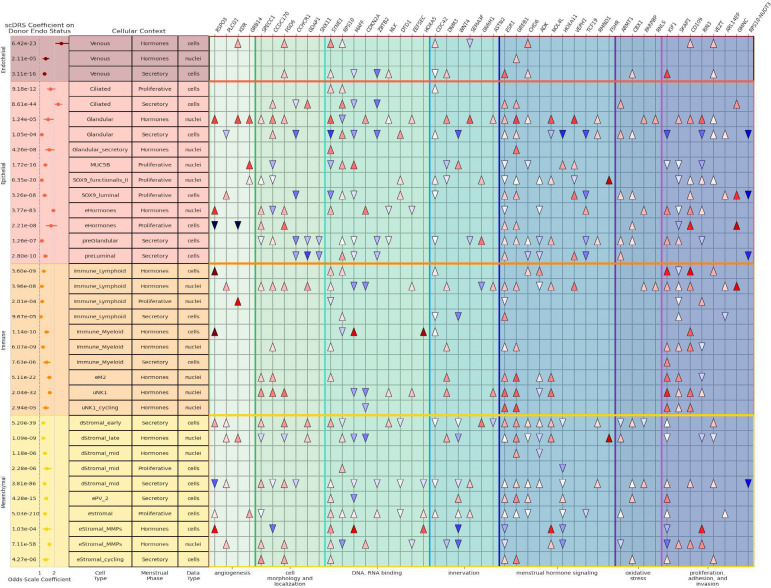
Single cell analyses – disease relevance scores (scDRSs) and differential gene expression. The left warm-colored panels show the odd-scale regression coefficients when comparing cells from donors with endometriosis to donors without endometriosis. Each row is annotated with the menstrual stage, cell type, and data type. The rows are ordered by cell type lineage. The right cool-colored panels show the results of two-sided t-tests comparing the gene expression of the top MAGMA genes, with significant tests represented by upward tringles for overexpression and downward triangles for underexpression.

We prioritized 34 cellular contexts (cell type – menstrual stage pairs) with significant regression coefficients (P < 2.08 × 10^−4^) for comparison of gene expression among the top MAGMA-identified genes (Figure 3). Regression results of endometriosis status on single cell disease risk score (scDRS) value across all tested cell type-menstrual stage pairs are provided in Supplementary Table 13.

Of the 34 cell contexts with significant regression effect estimates, 14 were derived from donors in the “hormones” menstrual phase, five from the proliferative phase, and 15 from the secretory phase. These contexts spanned all four cellular lineage groups: three endothelial, eight epithelial, ten immune, and 13 mesenchymal. The largest regression coefficient was observed in venous cells from donors on hormones (odds-scale coefficient = 2.34, P = 1.02 × 10^−18^). The most statistically robust signal emerged in endometrial stromal cells sampled during the proliferative phase (odds-scale coefficient = 1.40, P = 1.58 × 10^−231^). Among the genes driving the performance of each regression model, *ESR1* was most frequently differentially expressed, reaching significance in 24 of the cellular contexts, with 19 of those being overexpression and 5 demonstrating depletion. *GREB1* was the most consistently upregulated gene (23 contexts: 10 hormones, 10 secretory, and 3 proliferative), whereas *ADK* and *ZBTB2* were the most consistently downregulated (7 contexts each). Decidualized stromal cells from the mid-secretory phase exhibited the largest number of differentially expressed genes overall (21 genes: 13 upregulated and 8 downregulated).

Eight differentially expressed genes overlap with seven of GWAS loci that had not been previously associated with endometriosis. Gene expression results across all cell type–menstrual stage contexts are provided in Supplementary Table 14. *FSHR* demonstrated strong overexpression in two nucleus types: proliferative-phase SOX9 functionalis II (11.8-fold, P = 1.79 × 10^−7^) and late decidualized stromal from donors on hormones (11.0-fold, P = 1.36 × 10^−22^). Menstrual-stage-dependent association directions were observed for *EEFSEC*; in particular, uterine natural killer nuclei had a higher expression of *EEFSEC* gene in donors taking exogenous hormones (1.52-fold, P = 3.5 × 10^−12^), but in the same type of nuclei in the secretory phase, it was reduced (0.702-fold, P = 1.50 × 10^−9^). Two immune nucleus types from donors taking hormones showed high enrichment of *GMNC*, lymphoid (7.99-fold, P = 4.1 × 10^−8^) and myeloid (6.02-fold, P = 2.45 × 10^−5^). Relative expression of *RSPO3* within stromal cells peaked during the early secretory phase (1.78-fold, P = 1.65 × 10^−14^) relative to the proliferative stage (1.17-fold, P = 1.79 × 10^−8^) and the mid secretory phase (0.325-fold, P = 5.00 × 10^−133^). Two genes from the *ADK* locus, *AP3M1* and *ADK* shared associations with two cellular contexts, late-secretory decidualized stromal cells and pre-luminal secretory cells. *DTD1* was differentially expressed in five stromal cell contexts, four positively (early, mid, late decidualized and proliferative nondecidualized) and one negatively (nondecidualized secretory-stage stromal cells: 0.91-fold, P = 3.84 × 10^−13^). Six of the ten *NOL4L-*associated contexts involved nucleus types from donors exposed to exogenous hormones, all demonstrating positive association (1.46- to 2.72-fold, P < 1.36 × 10^−5^).

### Post-GWAS Multi-Omic Analyses Fuel Pathway Identification

**Figure 4:**
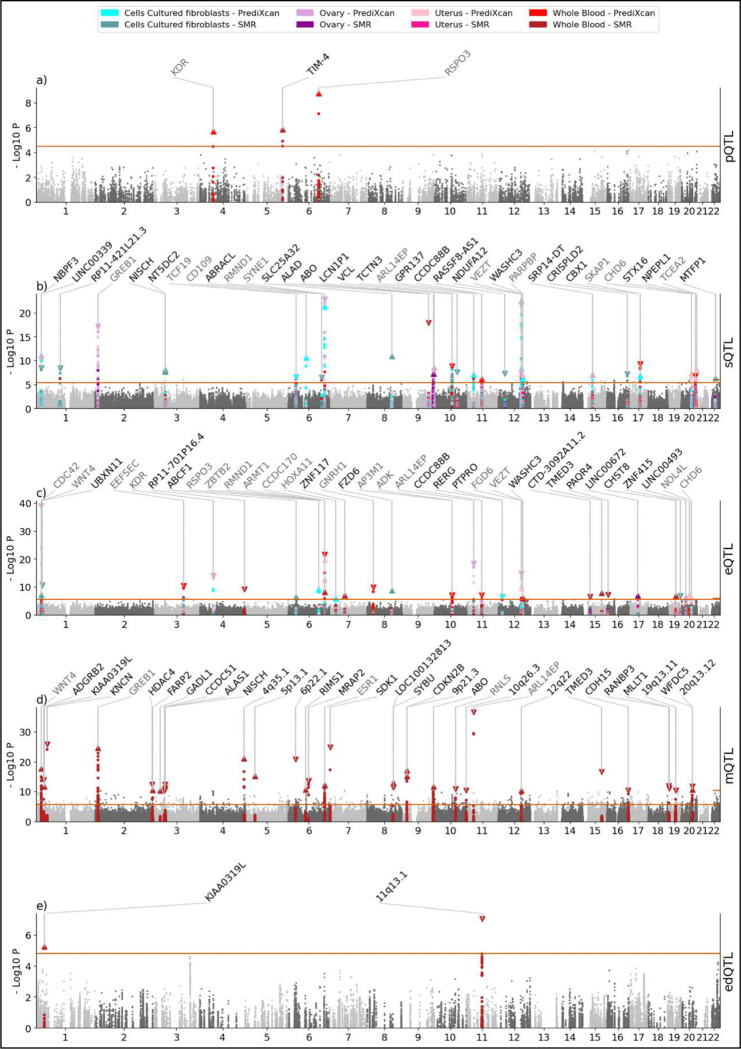
xQTL summary-based Mendelian randomization (xSMR) and PrediXcan imputed “x”ome-wide association study (xWAS) results across four tissue types and five -omic layers: a) proteome, b) splice-ome, c) transcriptome, d) methylome, and e) RNA edit-ome. The p-value thresholds were adjusted for the numbers of tests per analysis (count of sites per combination of GWAS – QTL dataset). The number of genes displayed on each panel is limited to the 35 most significant. Genes annotated in gray overlap with MAGMA genes whereas genes annotated in black do not. Triangles point upward for a positive effect and down for a negative effect.

To identify associations between endometriosis and additional layers of the central dogma and beyond (p - proteome, s - splice-ome, e - transcriptome, m - methylome, and ed - RNA edit-ome), we performed imputed association studies (xWAS) and QTL-wide summary-based Mendelian randomization (SMR), inputting the eleven GWASs’ summary statistics (See Methods). In total, xWAS implicated 54 unique molecular phenotypes, including 2 proteins, 73 introns, and 35 transcripts (Supplementary Table 15). Causal relationships involving 235 unique genes were identified by SMR, represented by 1 pQTL, 57 sQTLs, 34 eQTLs, 353 mQTLs, and 6 edQTLs (Figure 4; Supplementary Table 16).

Of the 266 genes implicated by xQTL summary-based Mendelian randomization (xSMR) and PrediXcan imputed “x”ome-wide association studies (xWAS), 32 overlapped with genome-level associations identified by MAGMA. Above and beyond the GWAS-related genes, pSMR and PWAS detected one protein, sSMR and SWAS identified introns at 33 genes, eSMR and TWAS revealed 44 genes, mSMR identified methylation sites at 164 genes, and edSMR implicated RNA editing sites at three genes. Several genes were supported by multiple layers of evidence, notably *KDR* and *RSPO3* which exhibited associations across MAGMA, the proteome, and the transcriptome. In contrast, we detected associations with *CCDC88B* and *WASHC3 in* both the splice-ome and transcriptome, but no corresponding associations were detected at the genome-level by MAGMA.

The only pSMR signal without significant heterogeneity was the protein TIM-4, with a positive association across three GWASs: AMR W (B = 0.594, P_SMR_ = 3.26 × 10^−5^), META PCN.v1 (B = 0.402, P_SMR_ = 1.48 × 10^−6^), and META PCN.v2 (B = 0.384, P_SMR_ = 1.27 × 10^−5^). *VCL* was the only gene in the intersection of the sSMR with SWAS results, represented by three distinct splicing events with associations across all four tissues, chr10:74109156–74111909 (B = −0.0803, P_SMR_ = 2.81 × 10^−7^, P_SWAS_ = 1.32 × 10^−9^), chr10:74109156–74114184 (B = 0.0692, P_SMR_ = 8.37 × 10^−8^, P_SWAS_ = 2.93 × 10^−9^), and chr10:74112112–74114184 (B = −0.0735, P_SMR_ = 1.51 × 10^−6^, P_SWAS_ = 4.52 × 10^−8^). The most-replicated transcriptome result was the association of endometriosis with reduced *EEFSEC* expression in both whole blood and cultured fibroblast cells (min P_SMR_ = 5.51 × 10^−7^ and min P_TWAS_ = 4.31 × 10^−11^). The gene with the highest number of significant CpG sites (12) from mSMR was *ESR1*. Methylation sites from intron 1 had positive SMR coefficients (highest B = 0.197, P_SMR_ = 2.16 × 10^−7^; probe cg04211581) whereas methylation sites from intron 2 had negative SMR coefficients (lowest B = −0.283, P_SMR_ = 3.02 × 10^−7^; probe cg24764793). Of the six significant RNA-editing sites, the most robust site (chr3:156540497) showed six significant edSMR associations across three tissues. This site in the *SSR3* gene demonstrated stronger effect sizes with the SCN.v1 (surgically-confirmed) GWAS than with the wide phenotype (B = −0.0769, P_SMR_ = 2.61 × 10^−5^ for fibroblast cells and B = −0.0731, P_SMR_ = 3.06 × 10^−5^ for uterus).

Including genomic signals from MAGMA, we identified 282 genes across six -omics types, each supported by respective biological evidence of endometriosis association. We performed chi-squared tests for this gene list with gene sets curated by MSIGDB (Supplementary Table 17) and identified several significant groups. From the Biocarta category of gene sets, there was only one gene set identified, the CREM pathway (P = 1.66 × 10^−7^). Top regulatory gene sets include target genes of *MIR128_1_5P* (P = 9.68 × 10^−11^), *ZNF92* (P = 7.70 × 10^−10^), and *MIR124_5P* (P = 1.78 × 10^−7^). The most significant biological processes from the GO (gene ontology) were sinoatrial node cell development (P = 3.44 × 10^−8^), mitochondrial fission (P = 6.99 × 10^−8^), and regulation of DNA ligation (P = 6.08 × 10^−7^). The strongest hit from the GO molecular function category was Wnt protein binding (P = 5.89 × 10^−10^).

## Discussion

We report the most diverse GWAS of over a million women for endometriosis to date incorporating participants from 14 biobanks across the world. Out of 58 loci from 11 meta-analyses, 29 are previously unreported (20 W, 3 Wex, 6 PCN and SCN) while 26 are in LD with (R^2^ > 0.1) and three more are proximal to (1 Mb) known signals. We have specified biological evidence of the genes or proteins relevant to 15 of the previously unreported loci. In addition to specifying 85 putative causal variants, we also detected shared genetic architecture between endometriosis and 135 complex polygenic phenotypes. Then, with the integration of five additional -omics layers, we have implicated a total of 282 genes associated with endometriosis.

Our ancestry-stratified meta-analyses revealed a consistent observed SNP heritability of 9–13% for endometriosis for four studied genetic ancestry populations with sufficient size (AFR, AMR, EAS, and EUR), which is higher than previously observed SNP heritability of 7% based on a majority-European population^6^. The higher and more consistent heritability we observed can be attributed to the unprecedented scale and diversity of our study cohort. The previous GWASs have not included any African-ancestry (AFR), Admixed-American-ancestry (AMR), or Central/South Asian (CSA+SAS) populations, which were essential in the computation of 12 / 13 (92%) credible sets in our fine-mapping analysis. We detected two multi-ancestry loci whose associations were driven by the AFR cohorts (they were only also significant in the AFR meta-analysis): *2p13.3* and *20q13.2*. By incorporating biobanks from various regions and ancestry backgrounds worldwide, we were able to capture a more comprehensive genetic landscape of endometriosis.

Given that diagnosis codes from the EHR can lack sensitivity for accurately phenotyping endometriosis^29^, we employed narrow phenotyping algorithms which incorporated procedure codes (surgical and imaging). Despite the diminished sample sizes, there were still 13 genome-wide significant loci: eight for Wex, four for PCN.v1, two for PCN.v2, and one for SCN.v1 (*21q22.13* is shared between PCN.v1 and SCN.v1). Four of the loci from the more precise phenotypes were successfully fine-mapped, including *FSHR:1* for Wex and *19q12* for PCN.v1, with one and seven putative causal variants in their credible sets, respectively. MAGMA detected regional associations with three genes (*LRRC2* with PCN.v1 and *GLB1L3* and *TCEA2* with Wex) that were not detected in the largest GWAS. Leveraging multi-modal EHR data for more precise phenotyping enhanced our ability to refine and validate genetic associations and uncover deeper biological insights.

Endometriosis has multifaceted risk factors and complex clinical presentations with a wide range of comorbidities and concomitant conditions. We explored genetic correlation and pleiotropy with endometriosis on an unprecedented scale (3,065 tests), leveraging phenome-wide summary statistics from All of Us and the UK Biobank along with metabolite, microbiome, and complex proteome GWASs. With the addition of Mendelian randomization, we have yielded invaluable hypothesis-generating results for further validation, even if genetic variants do not always meet the requisite instrumental variable assumptions for detecting causality. Five metabolites showed nominally significant (p < 0.05) genetic correlation with endometriosis, including serotonin which is known to be related to endometrial stromal cell decidualization^30^ but is also impacted by SSRIs like citalopram which may improve endometriosis symptoms^31^. We have observed vertical pleiotropy supporting a potential causal relationship between endometriosis and the reduction of the gut microbiome family *bifidobacteriaciae* (order *bifidobacteriales*, class *actinobacteria*), which plays a role in estrogen regulation^32,33^.

Results from across our multi-omic analyses underscore the potential role of R-spondin 3 (*RSPO3*) in endometriosis pathophysiology as a previously-characterized promotor of fibrotic proliferation^34^. *RSPO3*, known to modulate risk of endometriosis^35^, interacts with *WNT4* via Frizzled (*FZD*) receptors, influencing the WNT/Ca2+ and WNT/β-catenin pathways^36^. Wnt protein binding (GOMF) was a significant pathway in our gene set analyses, with five genes overlapping with our biologically-implicated endometriosis gene set: *CTHRC1* (mSMR), *FZD6* (fibroblast-eSMR), *PTPRO* (fibroblast-, ovary-, and whole blood-TWAS), *RECK* (fibroblast-sSMR), and *TRABD2A* (mSMR). Specifically, within the Wnt protein family, evidence for *WNT4’s* inverse association with endometriosis is found in six endometrial cellular contexts, mSMR (positive coefficient) and uterus- and ovary-TWAS (negative coefficient); methylation suppresses gene expression. *WNT4* interacts with *ESR1* (Estrogen Receptor Alpha), key regulator of endometrial cell proliferation and survival throughout the menstrual cycle^37^. *ESR1* was positively associated with endometriosis in 19 endometrial cellular contexts, and mSMR returned causal CpG site associations with intron-dependent effect directions. There were three stromal cellular contexts in which the expressions of *RSPO3*, *WNT4*, and *ESR1* were significantly different depending on endometriosis status. During the proliferative phase, all three were upregulated in nondecidualized stromal cells from donors with endometriosis; in the early secretory phase, *WNT4* switched to being downregulated, and in the mid secretory phase, both *WNT4* and *RSPO3* were diminished. Uncovering the interplay between these key genes throughout the menstrual cycle will be essential for understanding endometriosis pathogenesis.

One hallmark of endometriotic lesions is cellular adhesion, the process by which cells migrate and interact with the extracellular matrix^38,39^. An essential cytoskeletal protein which plays a role in regulating adhesion and motility is vinculin (Vcn)^40^. We detected three endometriosis-associated splicing events via sSMR and SWAS which support that expression of an isoform of Vcn, metavinculin (MVcn, +exon 19), may reduce endometriosis risk. The AA genotype at rs7896966 is associated with increased inclusion of exon 19^41^, and our GWAS identified that the AA genotype is also associated with decreased rates of endometriosis. It has been demonstrated that MVcn expression contributes to fewer but larger focal adhesions per cell^40^ and that the inhibition of focal adhesion via FAK decreases the formation of endometriotic lesions in mice^39^. While the GO gene set for focal adhesion assembly was not statistically significant, there were four genes that overlapped with that pathway: *VCL* (sSMR), *WNT4* (mSMR, uterus- and ovary-TWAS), *RHOD* (fibroblast-, uterus-, and ovary-eSMR), and *KDR* (six cellular contexts, mSMR, PWAS, and fibroblast-, uterus-, and ovary-TWAS). *RHOD* expression (codes for the GTPase RhoD) had a negative causal relationship with endometriosis, which aligns with previously published results that show that silencing RhoD leads to less efficient cell migration^42^. *KDR* exhibited mixed effect directions. Plasma PWAS along with endothelial cells and decidualized stromal cells supported positive associations whereas TWAS and stromal matrix metalloproteinase cells from the secretory phase indicated a negative association with *KDR* expression. When KDR (a.k.a. vascular endothelial growth factor receptor 2; *VEGFR2*) is stimulated by vascular endothelial growth factor (VEGF), it stimulates DNA synthesis and promotes focal-adhesion-related cell migration^43^. Although more investigation is required to precisely elucidate how the proteins in this pathway interact in the context of endometriosis, our data clearly support the hypothesis that the regulation of focal adhesion may be a strong candidate for therapeutic targets^39^.

Given the strong interplay between endometriosis and regulation of the menstrual cycle, it is important to take note of genetic and molecular signatures which may indicate hormonal dysregulation. *GREB1* (codes for Growth Regulating Estrogen Receptor Binding 1) is a well-characterized endometriosis-associated gene. We identified 23 cellular contexts in which *GREB1* expression is elevated in cells from donors with endometriosis, mostly from the cells and nuclei sampled during the secretory phase and from donors exposed to exogenous hormones. All five associations between elevated *GREB1* and endometriosis under the immune lineage were in nucleus types from donors on hormones. Since hormones are a common first-line treatment of endometriosis symptoms^44^, it is important to investigate how these medications interact with immune cell types that contribute to an inflammatory response. We have also replicated associations with known methylation and splice sites at the *GREB1* locus^45^, while also expanding upon the multi-omic molecular signature. *GREB1* has a truncated isoform, GREB1c, and we demonstrate that splicing events in ovarian and uterine tissue associated with this isoform increase risk of endometriosis while splicing events associated with the canonical isoform (GREB1a) are associated with decreased disease risk. Importantly, *GREB1* was not associated with endometriosis on the eQTL or pQTL levels, emphasizing that in order to promote mechanistic hypotheses of endometriosis, we must continue to incorporate comprehensive layers of multi-omics.

Despite the large sample size, heritability observed with GWAS (10–13%) still fails to measure up to the broad sense heritability estimation from a twin study of 47%^9^. Rare variants, structural variants, nonlinear effects, or gene-environment interactions might contribute to endometriosis risk but remain undetected in our analyses. As we leveraged the GWAS results to study other -omics from the central dogma and beyond, one limitation was that we lacked individual-level data. The results captured by xWAS and xSMR relied on previously-trained *cis*-xQTL models that are limited to capturing only genotype-driven molecular trait variability. This may leave out complex genetic architecture (*i.e. trans*-xQTLs) or environmentally-driven molecular signatures potentially relevant to our phenotype of interest^46^. While our innovative and comprehensive *in silico* multi-omic approach yielded many testable hypotheses, we did not perform any experimental functional validation. Furthermore, although our GWAS constitutes the most diverse genetic study of endometriosis performed so far (32% non-European), we still lacked the power to draw many meaningful conclusions in understudied populations. We want to emphasize that the field should continue prioritizing the inclusion of diverse cohorts in genetic studies.

In addition to replicating findings from previous studies, we uncovered new insights that advanced our understanding of the genetic underpinnings of endometriosis. Our integrative multi-omics approach, combining genomic data with single-cell analyses along with proteomic, splice-omic, transcriptomic, methylomic, and RNA edit-omic tests, has biologically implicated 282 genes, providing unprecedented insights into the molecular mechanisms driving this chronic condition. These findings lay a robust foundation for future functional studies to elucidate the precise roles of identified genes and pathways in endometriosis pathogenesis. Moreover, our results have important clinical implications, potentially informing the development of more accurate diagnostic tools, personalized risk prediction models, and targeted therapeutic interventions. As we move forward, this work emphasizes the critical importance of large-scale, diverse genetic studies in unraveling the complexities of multifactorial diseases like endometriosis, paving the way for improved patient outcomes and a deeper understanding of women’s health issues globally.

## Methods

### Phenotyping

Endometriosis diagnosis requires direct observation of lesions by laparoscopic surgery or sometimes imaging (MRI/ultrasound). We can extract diagnoses from medical records via diagnosis billing codes in the form of ontologies like the international classification of diseases (ICD) or the systematized nomenclature of medicine (SNOMED). For the EHR-linked biobanks, we used structured data to define the phenotypes. Cases for wide endometriosis (W) were any women with a history of ICD-9 (617.*), ICD-10 (N80.*), or SNOMED codes for endometriosis. For the wide phenotype excluding adenomyosis (Wex), women with a history of ICD-9 (617.0), ICD-10 (N80.0), or SNOMED codes for uterine endometriosis were excluded from both cases and controls. Our two narrow case definitions were procedure-confirmed and surgically-confirmed (PCN and SCN). These narrow phenotypes were designed to capture confirmed cases and controls of endometriosis based on procedure history including hysterectomies, laparoscopies and ultrasounds. The list of procedure codes (CPT-4 or OPCS) for PCN included all the surgery codes in SCN but added non-obstetric ultrasound codes to account for potential imaging diagnoses. Each of the two narrow phenotypes was tested in two versions: cases versus all controls (PCN.v1 and SCN.v1) and cases versus confirmed controls (PCN.v2 and SCN.v2) where confirmed controls were those who had history of the corresponding procedure codes for each phenotype with no history of endometriosis diagnosis. For additional details on the phenotyping algorithms, see Supplemental Appendix 1.

### Genotyping by Biobank

Most contributing biobanks genotyped several hundred thousand variants. After those data are collected, then the rest of the genomic variants can be imputed probabilistically based on a reference panel such as TOPMED. Some biobanks such as AOU used short read whole genome sequencing (WGS) to gather their genomic data. The sequences from the short reads are aligned and compared to the reference to produce variant calls. References and platform information for each biobank’s genotyping method and QC can be found in Supplementary Table 18.

### Genetic Association Testing

Each contributing biobank performed ancestry-stratified association testing with up to six phenotypes (W, Wex, PCN.v1, PCN.v2, SCN.v1, and SCN.v2), depending on whether they had at least 50 cases for that phenotype and ancestry combination. Genetically-informed ancestry (GIA) is defined based on genetic distance from subpopulations of a reference group such as the 1000 Genomes Project dataset^47^ (1KG). Genetic distance is measured using a dimensionality reduction technique such as principal component analysis (PCA). Ancestry is then assigned using a classifier such as a mixture model or k-nearest neighbors. For references and methods used for assigning GIA within each biobank, see Supplementary Table 19.

Contributing biobanks used linear mixed models to estimate the effect of each variant on the six phenotypes. Tools implemented for these tests include SAIGE^48^ and Regenie^49^. Association tests were adjusted for principal components, age at EHR data collection, and any biobank-specific batch variables (for example, collection site within eMERGE). For estimation of the null models (SAIGE or Regenie step 1), variants were required to have a call rate of at least 95%, a minor allele frequency of 0.01, and a Hardy-Weinberg p-value of at least 1×10^−6^. For association testing (SAIGE or Regenie step 2), variants were required to have a minor allele count of at least 20, and an imputation quality of at least 0.6. Individuals were included if their overall genotyping rate was at least 95%. A summary of each biobank’s exact covariates and QC procedures is in Supplementary Table 20. We leveraged publicly-available FinnGen^50^ summary statistics to increase the sample size.

After biobank analyses were completed and collected, summary statistics were cleaned and lifted into genome build hg38 as necessary. Then, inverse variance-weighted, fixed-effect meta-analyses were performed using GWAMA (software for genome-wide association meta-analysis)^51^. GWAMA adjusts each study based on its overall genomic inflation factor. Prior to meta-analysis, input summary statistics were restricted to variants with a minor allele frequency of at least 0.005 to ensure stability in the estimates.

To define significantly-associated loci, we started by applying the clumping function of Plink 1.9^52^ with the lead SNP p-value threshold set to 5×10^−8^, the secondary SNP threshold set to 1, minimum R^2^ of 0.1, and a window of 1000kb. 1KG was used as a linkage disequilibrium (LD) reference, matching the whole dataset to the multi-ancestry meta-analyses and single-ancestry subsets of the dataset to their respective meta-analyses. After the variant clumps were identified, any clumps that were physically overlapping with one another were merged into loci. Then, we tested if any loci were in LD with (R^2^ > 0.25) or proximal to tag SNPs of the 39 autosomal lead SNPs from the 2023 GWAS^6^ to determine whether our hits were known or previously unreported^16^. Each locus is labeled with its cytoband, but if the corresponding gene region was statistically significant in the MAGMA, xSMR, or xWAS tests (described below), or if the GWAS locus is in contact with a gene based on endometrial cell chromatin looping data^53^, that gene symbol was added to the locus label.

### Estimation of Observed SNP Heritability and Genetic Correlation Amongst Endometriosis GWASs

We estimated heritability with the LD-Score Regression tool, LDSC^54^, for all single-ancestry meta-analyses representing at least two thousand cases. LD scores were computed for HapMap variants^55^ from the respective super-population subgroups from 1KG for each ancestry-stratified meta-analysis. We also estimated partitioned heritability^56^ with variant annotation groups coming from ENSEMBL regulatory features GRCh38.105^57^ and uterus-specific regulatory features from EpiMap^58^. We estimated cross-ancestry genetic correlation of our meta-analyses using Popcorn^59^. Popcorn cross-population LD scores were derived from the same set of variants and samples from 1KG as in LD score computation for LD score regression.

### Statistical Fine-Mapping for Identification of Putative Causal Variants

The multi-ancestry sum of the single effects model (MESuSiE)^60^ was used to identify putative causal variants in each significant GWAS locus from the multi-ancestry meta-analyses. For each locus, we extracted the single-ancestry summary statistics from that region in addition to computing ancestry-specific LD matrices for AFR, AMR, EAS, EUR, and SAS. MESuSiE analyzes whether causal variants are shared between ancestry groups by computing a posterior inclusion probability (PIP) for each ancestry alone and for each combination of two or more ancestries. SNPs with any PIP value greater than 0.5 are part of the credible set for that locus.

### Variant-Set Enrichment for Identifying Enriched Gene Regions

MAGMA^61^ was used to assess gene regions within the GWAS results for enrichment. MAGMA uses a model based on multiple regression to test the association of a phenotype with groups of variants. Testing groups of variants increases statistical power over the single-variant GWAS tests performed^61^. We utilized gene region definitions from NCBI for human genome build 38, testing between 18,691–18,846 genes for each GWAS meta-analysis (MAGMA test counts and p-value thresholds are available in Supplementary Table 21).

### Phenome-Wide and Multi-Omic Genetic Correlation and Mendelian Randomization Tests

The AFR, EAS, and EUR wide phenotype meta-analyses were utilized for LDSC^54^ genetic correlation (GC) tests with phenome-wide GWAS summary statistics from AOU^62^ and UKB (EUR-only). In AOU, we tested for correlation with binary traits that had at least 2,000 cases (medications and phecodeX phenotypes) and quantitative traits with at least 2,000 samples overall (labs and biometric measurements), totaling 1,728 tests. From UKB, we utilized summary statistics for diagnoses and survey variables, both binary and quantitative, with heritability estimates for which the Z-score was at least 4 (527 studies)^63^. Summary statistics were also obtained for 169 metabolites^64^, 165 gut microbiome taxa^65^, and 487 proteins^66^ which we were unable to map to one genomic location (mappable proteins were utilized in SMR described below). Reference LD scores were computed from the corresponding 1KG superpopulation groups across Hapmap variants^55^. For the larger-sample tests (AOU and UKBB phenome), genetic correlation results with a p-value of < 0.05 were candidates for mendelian randomization, and for the smaller tests (proteome and microbiome), traits with an h^2^ p-value or rg p-value < 0.05 were nominated. Effect estimates were not available for the metabolites, so we could not perform MR on those. Significant GC estimates were those that reached a p-value < 1.63 × 10^−5^ (correcting for 3,065 total tests).

To distinguish horizontal and vertical pleiotropy, we performed secondary mendelian randomization (MR) tests on the 890 nominated traits using the MendelianRandomization R package^67^. Several MR methods were utilized as a sensitivity analysis for the estimates: inverse-variance weighted (IVW), debiased IVW, lasso-penalized IVW, weighted median, maximum likelihood, and MR-Egger (random-effects). We tested in both directions: endometriosis causing the test trait and vice-versa). Instrumental variables (IVs) were the genome-wide significant variants from the HapMap-filtered^55^ exposure summary statistics, and we used correlation matrices calculated with PLINK 2.0^68^ to adjust for the covariance between IVs. Significant causal relationships were those for which the maximum p-value across methods was < 0.05, the minimum p-value was < 2.84 × 10^−5^ (1,760 total bidirectional tests), all estimates were the same direction, and that direction matched the direction of the genetic correlation estimate.

### Integration with Single-Cell Data

Single cell data are rich resources for understanding the biology of diseases. A recently published endometrial single cell atlas includes gene expression data for cells, nuclei, immune cells, and immune nuclei, covering over 40 different cell types, three menstrual phases, and four different endometrial pathologies^69^. We leveraged these data to compute single-cell disease-relevance scores (scDRSs) based on the associated genes from GWAS, as computed by MAGMA. Disease risk scores were computed and normalized using the scDRS software^70^.

For each cell type and menstrual phase (“cellular context”), we computed the logistic regression coefficient of the scDRS comparing cells from donors with and without endometriosis. These regression models were adjusted for age; one source dataset only had an age range (18–36), so we assigned age with a random uniform distribution to those cells. These regression coefficients were used to prioritize cellular contexts (cell type – menstrual stage pairs). Significant contexts were those which had a coefficient one-sided p-value of < 0.05 / 240 tests. For prioritized contexts, we compared the expression of the top MAGMA genes (34 genes) between case and control donors using t-tests to identify which genes were driving the distinguishing effect of the scDRS. Significant t-tests were those with a p-value < 0.05 / 1,190 (34 genes × 35 significant contexts).

### Multi-Omic Imputed Association Studies and Mendelian Randomization

We used GWAS summary statistics from the 11 meta-analyses to estimate the association between endometriosis, transcript expression (TWAS), protein expression (PWAS), and intron inclusion (SWAS). For TWAS and SWAS, we used precomputed eQTL and sQTL weights, respectively, from the PredictDB data repository (http://predictdb.org/) which represented *cis*-level associations in uterine tissue, ovarian tissue, whole blood, and cultured fibroblast cells. For PWAS, we used whole blood pQTL models from *Schubert et al*^71^ which are based on the SOMAscan assay data measured in multi-ethnic Trans-omics for Precision Medicine (TOPMed) Multi-omics pilot study. We used baseline models only (no covariates) which had a predictive correlation (rho) of at least 0.1 and a z-score p-value threshold of 0.05, ensuring that the association between the SNPs and protein expression was statistically significant. The xQTL models along with the GWAS summary statistics were the input for summary-based PrediXcan (S-PrediXcan)^72^. We used Bonferroni-correct p-value thresholds to identify statistically significant associations for each tissue study. The alpha value was 0.05 and the numbers of tests for each GWAS-tissue-QTL combination can be found in Supplementary Table S22. For labeling sQTLs, GTEx provided a mapping from introns to transcripts.

Summary-Based Mendelian Randomization (SMR) is a publicly-available softare tool which leverages uploaded GWAS summary statistics to detect pleiotropic associations between molecular phenotypes as the exposure and a trait of interest as the outcome^73^. We tested all combinations between the 11 GWASs and the following QTL datasets: GTEx eQTLs, sQTLs, and edQTLs for ovary, uterus, whole blood, and fibroblast cells, plasma pQTLs from the FENLAND study^66^, McRae whole blood mQTLs^74^, and Hatton blood mQTLs derived from EAS and EUR populations^75^. The QTL was the exposure with endometriosis being the outcome. Significant causal associations were identified as those with a p-value less than 0.05 / the number of tests for each GWAS-tissue-dataset combination. Then, significantly heterogeneous results (HEIDI P < 0.05 / the number of significant tests) were discarded. The test counts and the corrected p-value thresholds for the xSMR analyses are available in Supplemental Table S23.

Once we aggregated all genes resulting from MAGMA, xWAS, and xSMR, we performed basic gene set testing using chi-squared statistics comparing our gene list to each pathway’s list of genes. The gene sets were from MSIGDB and included hallmark pathways, cell type signatures, co-regulation pathways, and GO ontology pathways. The numbers of gene set tests corrected for are available in Supplementary Table S24.

## Supplementary Material

Supplementary Files

This is a list of supplementary files associated with this preprint. Click to download.

• supplementaltables.xlsx

## Figures and Tables

**Table 1:** All GWAS meta-analysis sample sizes. All six phenotypes had a multi-ancestry meta-analysis as well as single-ancestry meta-analyses for AFR, AMR, CSA+SAS, EAS, and EUR. There were not enough CSA+SAS or EAS studies to conduct meta-analyses for all the narrow phenotypes.

Phenotype	Ancestry	N	Cases	Prevalence
W	AFR	96,012	4,289	4.47%
	AMR	67,335	2,136	3.17%
	CSA+SAS	8,164	259	3.17%
	EAS	168,209	7,833	4.66%
	EUR	723,937	35,748	4.94%
	META	1,063,657	50,265	4.73%
Wex	AFR	92,202	1,575	1.71%
	AMR	66,219	1,020	1.54%
	CSA+SAS	5,066	62	1.22%
	EAS	86,667	1,584	1.83%
	EUR	364,896	8,021	2.20%
	META	615,050	12,262	1.99%
PCN.v1	AFR	70,959	1,152	1.62%
	AMR	58,802	750	1.28%
	EAS	78,999	155	0.20%
	EUR	317,481	4,053	1.28%
	META	526,241	6,110	1.16%
PCN.v2	AFR	12,958	1,152	8.89%
	AMR	9,472	750	7.92%
	EAS	1,539	155	10.07%
	EUR	56,134	4,053	7.22%
	META	80,103	6,110	7.63%
SCN.v1	AFR	70,308	501	0.71%
	AMR	58,477	425	0.73%
	EUR	284,932	2,573	0.90%
	META	413,717	3,499	0.85%
SCN.v2	AFR	1,912	501	26.20%
	AMR	2,291	425	18.55%
	EUR	14,147	2,573	18.19%
	META	18,350	3,499	19.07%

Phenotype abbreviations: W = wide, Wex = wide excluding adenomyosis, PCN = procedure-confirmed narrow, SCN = surgically-confirmed narrow, .v1 = versus all controls, .v2 = versus confirmed controls. Ancestry abbreviations: AFR = African, AMR = admixed American, CSA = central/south Asian, SAS = south Asian, EAS = east Asian, EUR = European.

**Table 2:** Credible sets of multi-ancestry GWAS loci. nine are for the W phenotype, two are for Wex, one is for PCN.vl, and one for PCN.v2. An “X” in the ancestry population columns denote whether that ancestry group was used for detecting any SNPs in the credible set (PIP > 0.5). Locus names match Figure 1.

Phenotype	Locus	# CS	AFR	AMR	EAS	EUR	SAS	Credible Set
W	*WNT4*	1			X	X		rs2807365
W	*1p13.2*	4			X	X	X	rs12030576, rs2982742, rs61810746, rs6328
W	*GREB1*	3		X	X	X		rs7578132, rs10929759, rs59129126
W	*FSHR:2*	2		X	X	X	X	rs1504175, rs1504188
W	*CD109*	1	X	X		X		rs77740740
W	*7p15.2*	1	X		X	X		rs1451385
W	*7p12.3*	2		X	X	X		rs10479868, rs11975261
W	*ADK*	2		X	X	X	X	rs4268450, rs11001059
W	*DTD1*	4			X	X	X	rs6081248, rs6081313, rs34478401, rs6045577
Wex	*FSHR:1*	1		X		X		rs2300440
Wex	*9p21.3:2*	3	X		X	X		rs1333054, rs10965274, rs10811669
PCN.v1	*19q12*	7	X			X		rs56124241, rs10411368, rs12981956, rs59841764, rs57255653, rs12973006, rs12972676
PCN.v2	*MEGF6*	1		X		X		rs76419782

Phenotype abbreviations: W = wide, Wex = wide excluding adenomyosis, PCN = procedure-confirmed narrow, .v1 = versus all controls, .v2 = versus confirmed controls. Ancestry abbreviations: AFR = African, AMR = admixed American, EAS = east Asian, EUR = European, SAS = south Asian.

## Data Availability

All data required to recreate figures are available in supplemental tables S1-S24. GWAS summary statistics will be uploaded to the GWAS catalog upon manuscript acceptance. The availability of individual-level data varies by biobank. AOU, eMERGE, and UKBB are available to researchers by application. The other biobanks (BBJ, BioVU, CCPM, FinnGen, GHI, HUNT, Latvian National Biobank, MGBB, MVP, PMBB, and TWB) only allow access to institutional researchers via their respective IRB protocols. Code written and run by the coordinating center (PMBB) is available in this github repository: https://github.com/Setia-Verma-Lab/guare_et_al_endometriosis_gwas_2025.
